# Supplementary material for: Clearance of defective muscle stem cells by senolytics restores myogenesis in myotonic dystrophy type 1
Source: Nat Commun. 2023 Jul 19;14:4033. doi: 10.1038/s41467-023-39663-3 (PMC10356779; doi:10.1038/s41467-023-39663-3)
Supplement: Supplementary file 1 — Supplementary information [file 41467_2023_39663_MOESM1_ESM.pdf]

SUPPLEMENTAL MATERIAL FOR

Clearance of defective muscle stem cells by senolytics restores myogenesis in myotonic dystrophy type 1.

Talita C. Conte, Gilberto Duran-Bishop, Zakaria Orfi, Inès Mokhtari, Alyson Deprez, Isabelle Côté, Thomas Molina, Taeyeon Kim, Lydia Tellier, Marie-Pier Roussel, Damien Maggiorani, Basma Benabdallah, Severine Leclerc, Lara Feulner, Ornella Pellerito, Jean Mathieu, Gregor Andelfinger, Cynthia Gagnon, Christian Beauséjour, Serge McGraw, Elise Duchesne, and Nicolas A. Dumont

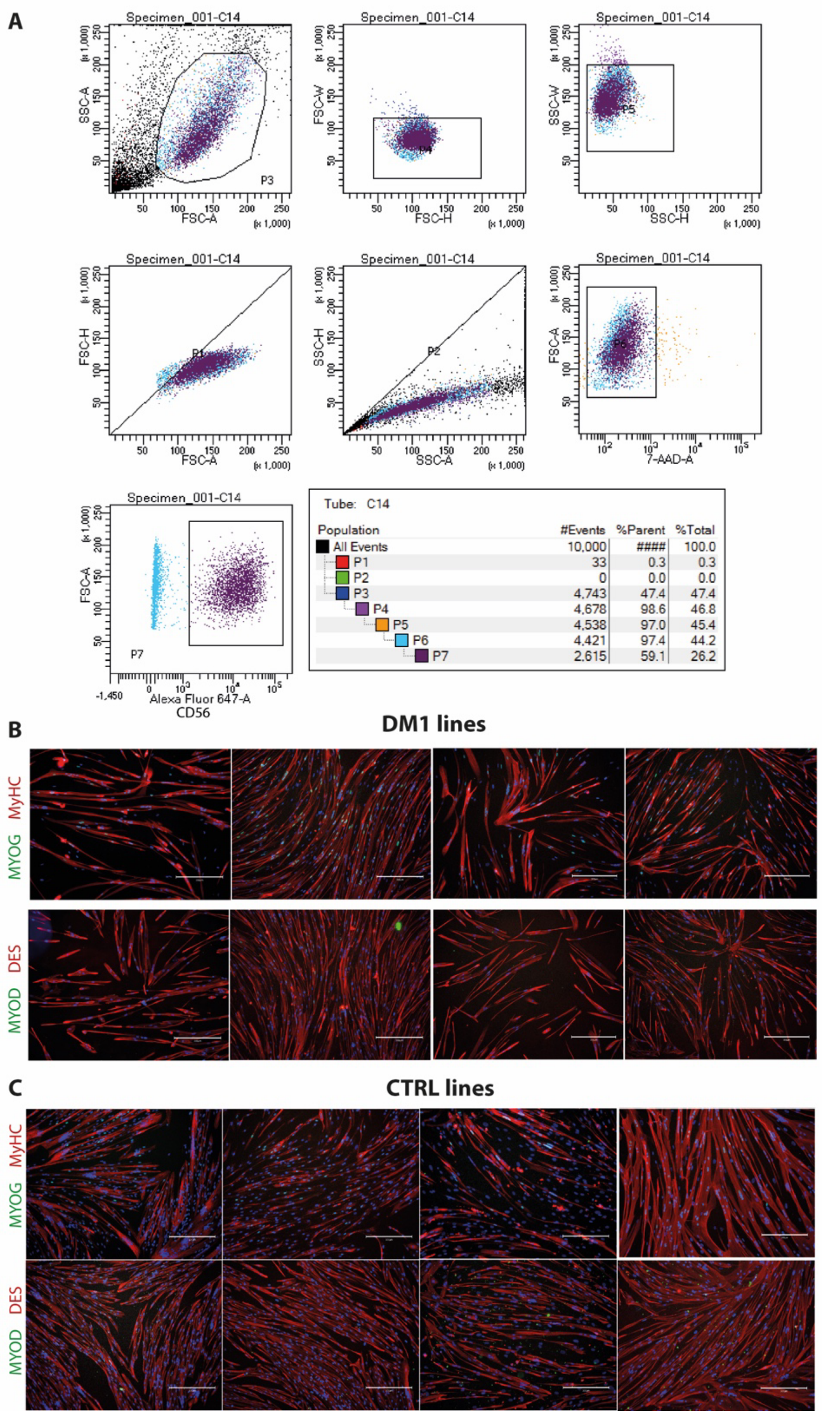

Supplemental Figure 1. Myogenic cell isolation by FACS. **A**) Representative FACS plot showing the gating strategy to purify human myogenic cells using forward scatter (FSC), side scatter (SSC), 7AAD (cell viability), and CD56 AF647-conjugated antibody (human myogenic cell marker). **B,C)** Representative micrographs of immunofluorescence for the myogenic markers Myogenin (Myog, green) and Myosin heavy chain (MyHC, red), or desmin (DES, red) and MYOD (green) on **B**) DM1 myoblasts and **C**) healthy control myoblasts. Scale bars = 300 µm. Experiments performed in duplicates with similar results.

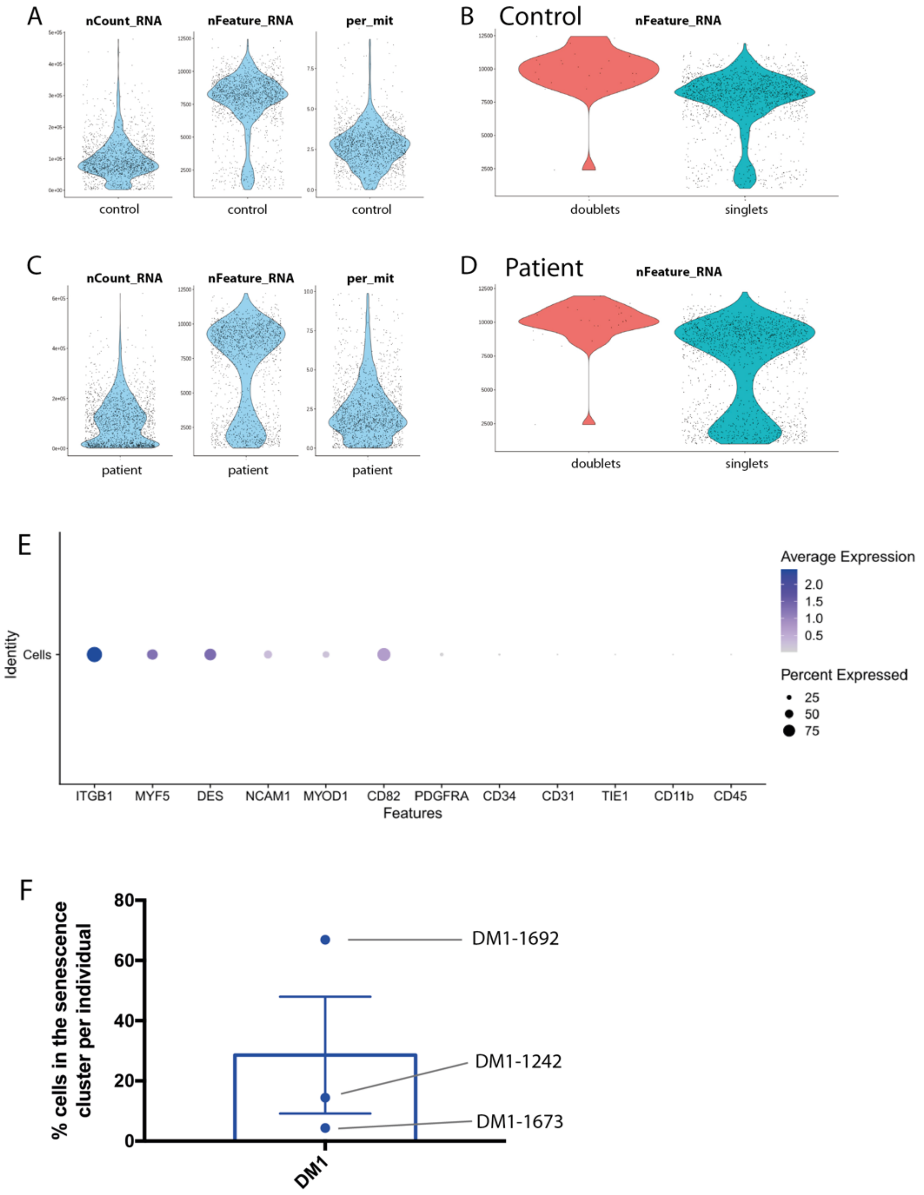

Supplemental Figure 2. Single cell RNAseq. A,C) Violin plots showing quality control features (nCount RNA, nFeature RNA, percent mito) and **B,D)** the identification of singlets vs doublets for healthy controls (**A,B**) and DM1 myoblasts (**C,D**) for the scRNAseq analysis. **E**) Dot plot showing the cell type markers for myogenic genes (*ITGB1, MYF5, DES, NCAM1, MYOD1, CD82*) and non-myogenic genes: fibroadipogenic progenitors (*PDGFRA, CD34*), endothelial cells (*CD31, TIE1*), and myeloid cells (*CD11B, CD45*) from the single-cell RNAseq dataset of DM1 patients and healthy controls. **F**) Percentage of cells per DM1 individuals that are clustering in the senescent cell subpopulation (n=3 biological samples). Data are presented as mean +/- SEM.

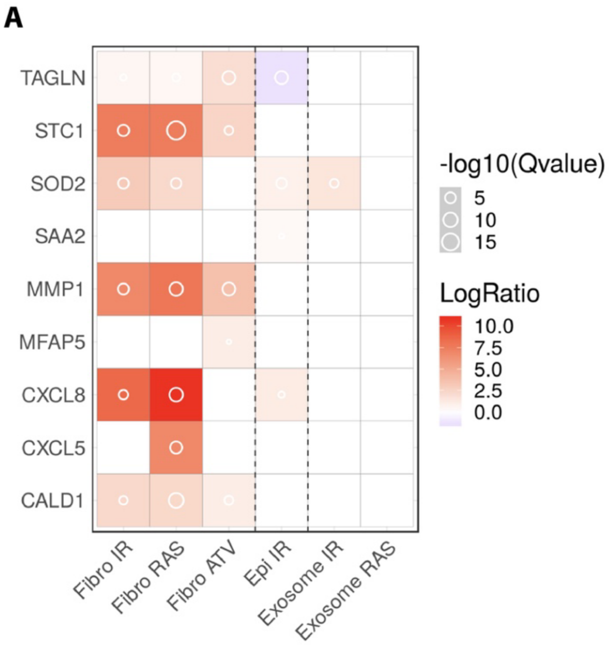

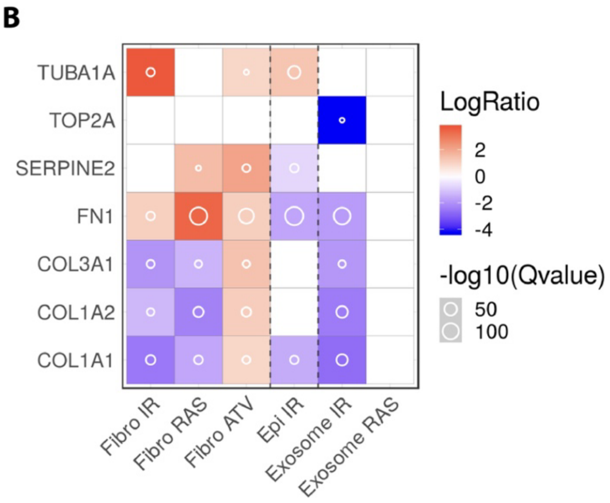

Supplemental Figure 3. **Expression of SASP in control and DM1 cells.** Heatmap comparing the genes overexpressed (>1.7 fold) in myoblasts from DM1 patients (**A**) and healthy controls (**B**) to the SASP Atlas (http://www.saspatlas.com/), a proteomic database of soluble proteins and exosomes of SASP factors secreted by different cell types: fibroblasts (Fibro) and epithelial cells (Epi) subjected to multiple senescence inducers: genotoxic stress-induced (IR; Irradiation), oncogene-induced (RAS overexpression), treatment-induced (ATV; Atazanivir). Red boxes indicate positive regulators of senescence that are overexpressed by senescent cells of the SASP Atlas. Blue boxes indicate negative regulators of senescence that are overexpressed in non-senescent control cells of the SASP Atlas.

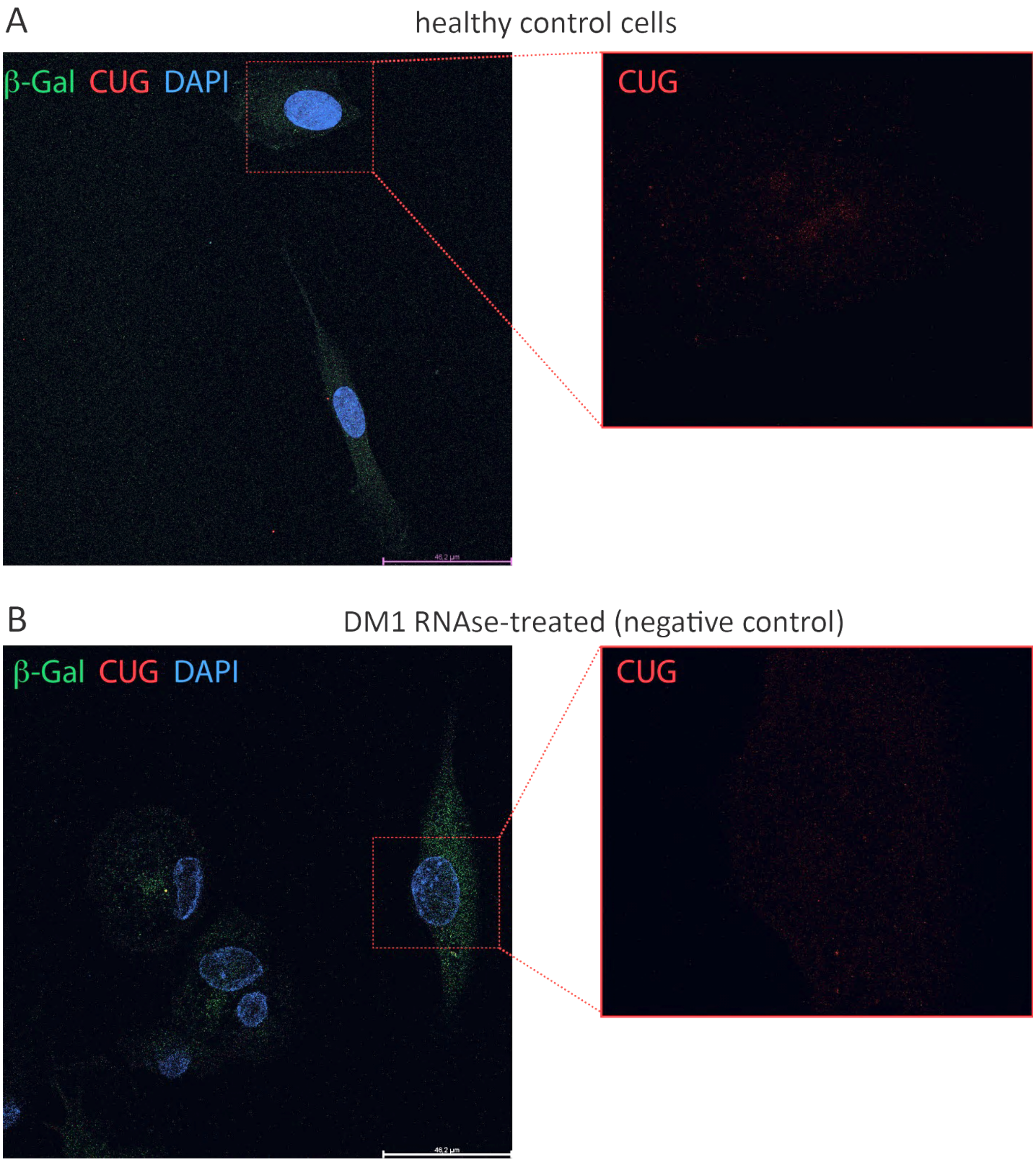

Supplemental Figure 4: Controls for RNA foci assessment. Representative micrograph of immunostaining of SA-β-Gal (green), CUG repeats (red), and DAPI (blue) for **A**) healthy control cells and **B**) DM1 myoblasts treated with RNAse as a negative control. Intranuclear RNA foci were not detected. Experiments performed in duplicates with similar results.

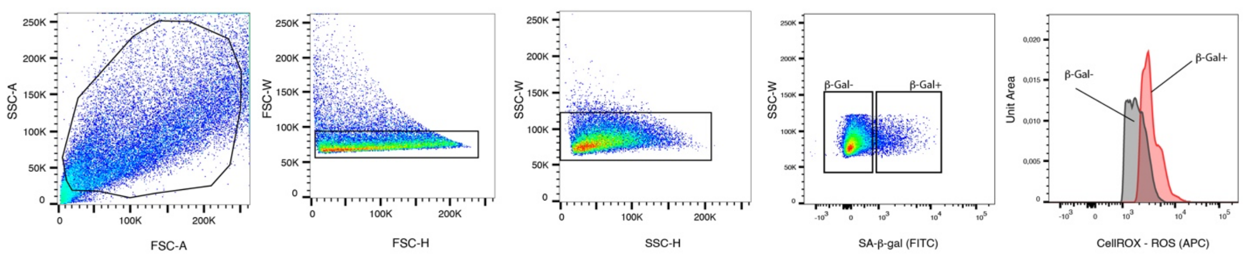

Supplemental Figure 5. ROS expression in senescent DM1 myoblasts. DM1 myoblasts were labeled with FITC-conjugated SA-β-Gal (senescence marker) and CellROX (ROS marker). After selection for forward scatter (FSC) and side scatter (SSC), the senescent (FITC-high) and non-senescent cells (FITC-low) were analyzed for the expression of ROS (CellROX, APC). Senescent cells express higher levels of ROS than non-senescent cells. The experiment was repeated independently twice (with different cell lines) with similar results.

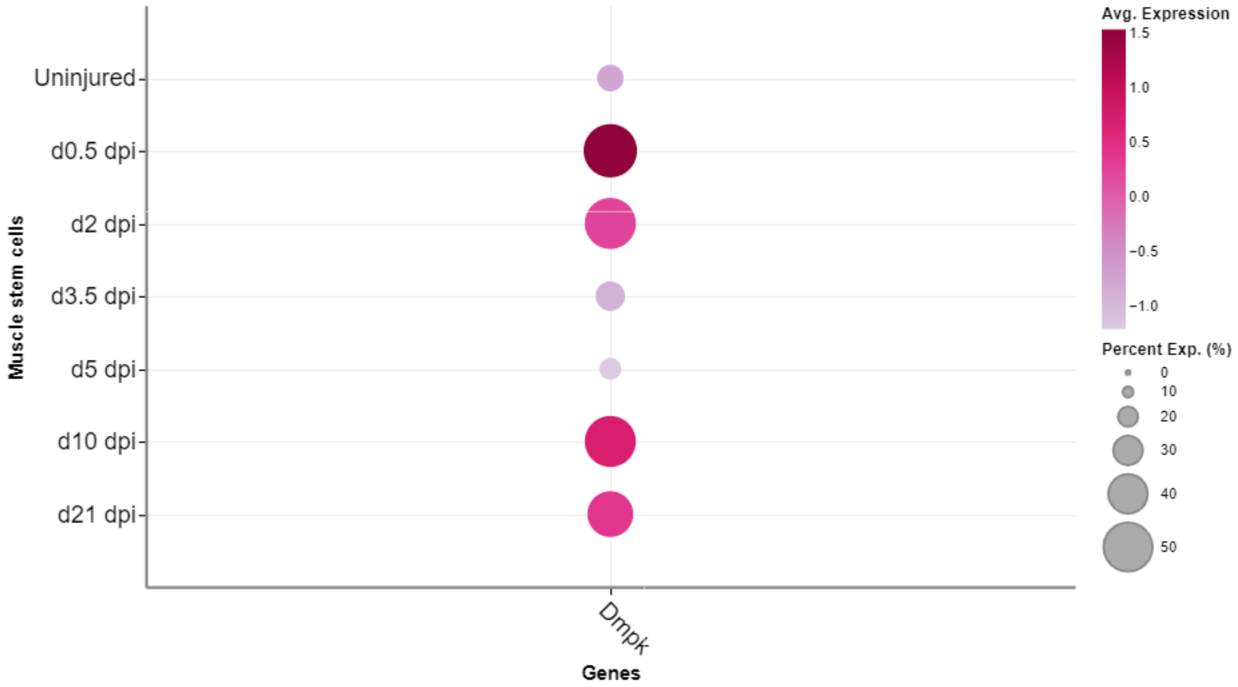

Supplemental Figure 6. *DMPK* expression during the different stages of myogenesis. Expression of *DMPK* in myogenic cells based on publicly available dataset (Oprescu SN et al, iScience 2020; DOI: 10.1016/j.isci.2020.100993) in which skeletal muscle were injured by cardiotoxin and cells were isolated at 0, 0.5, 2, 5, 10, and 21 days post-injury (dpi). These time points cover the different stages of myogenesis from MuSC quiescence, activation into myoblasts, proliferation, differentiation, fusion, and return to quiescence. The size of the dots represents the percentage of cells expressing the gene and the color of the dots represents its level of expression.

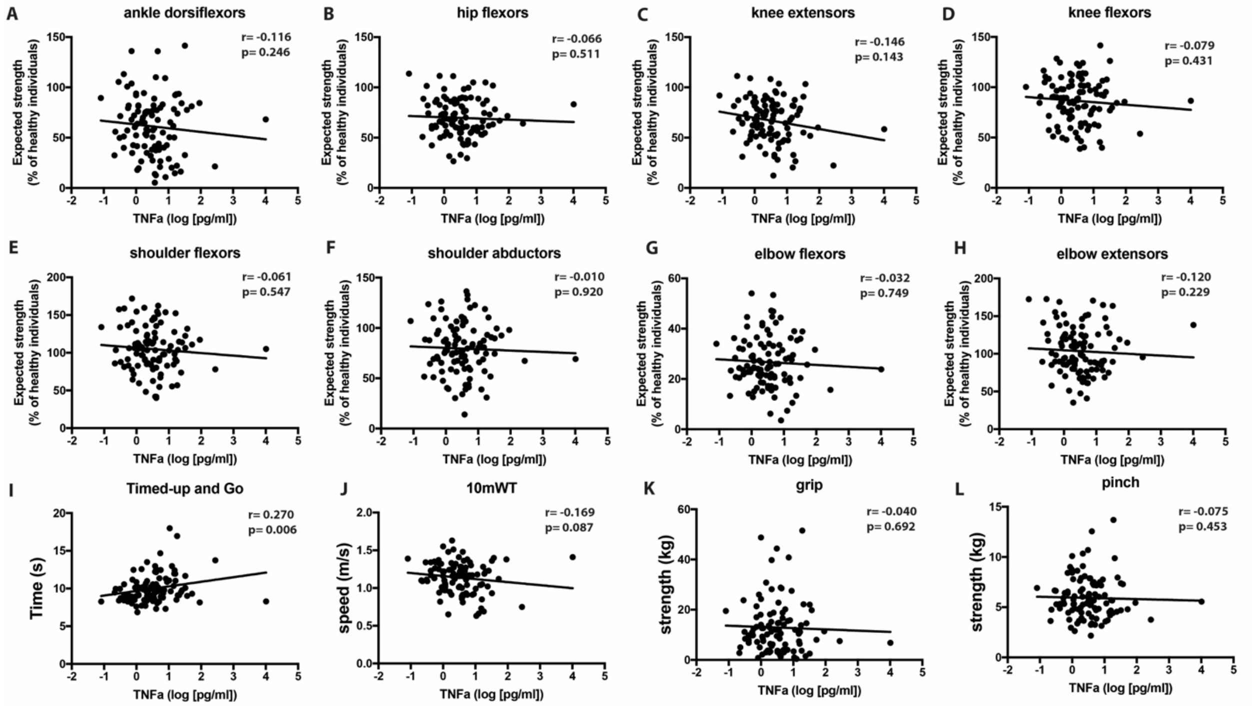

Supplemental Figure 7. TNFα level is not correlated with muscle strength and functional outcomes in DM1. Correlation between serum TNFα levels in DM1 patients and the expected strength (relative to normative values) of different muscle groups of **A-D**) the lower limb (ankle dorsiflexors, hip flexors, knee extensors, knee flexors), **E-H**) the upper limb (shoulder flexors, shoulder abductors, elbow flexors, elbow extensors) and **I-L**) different functional capacity tests (Timed-up and Go, 10-meter walk test; 10mWT, grip test, pinch test). N=103 patients. Spearman ρ correlation coefficient.

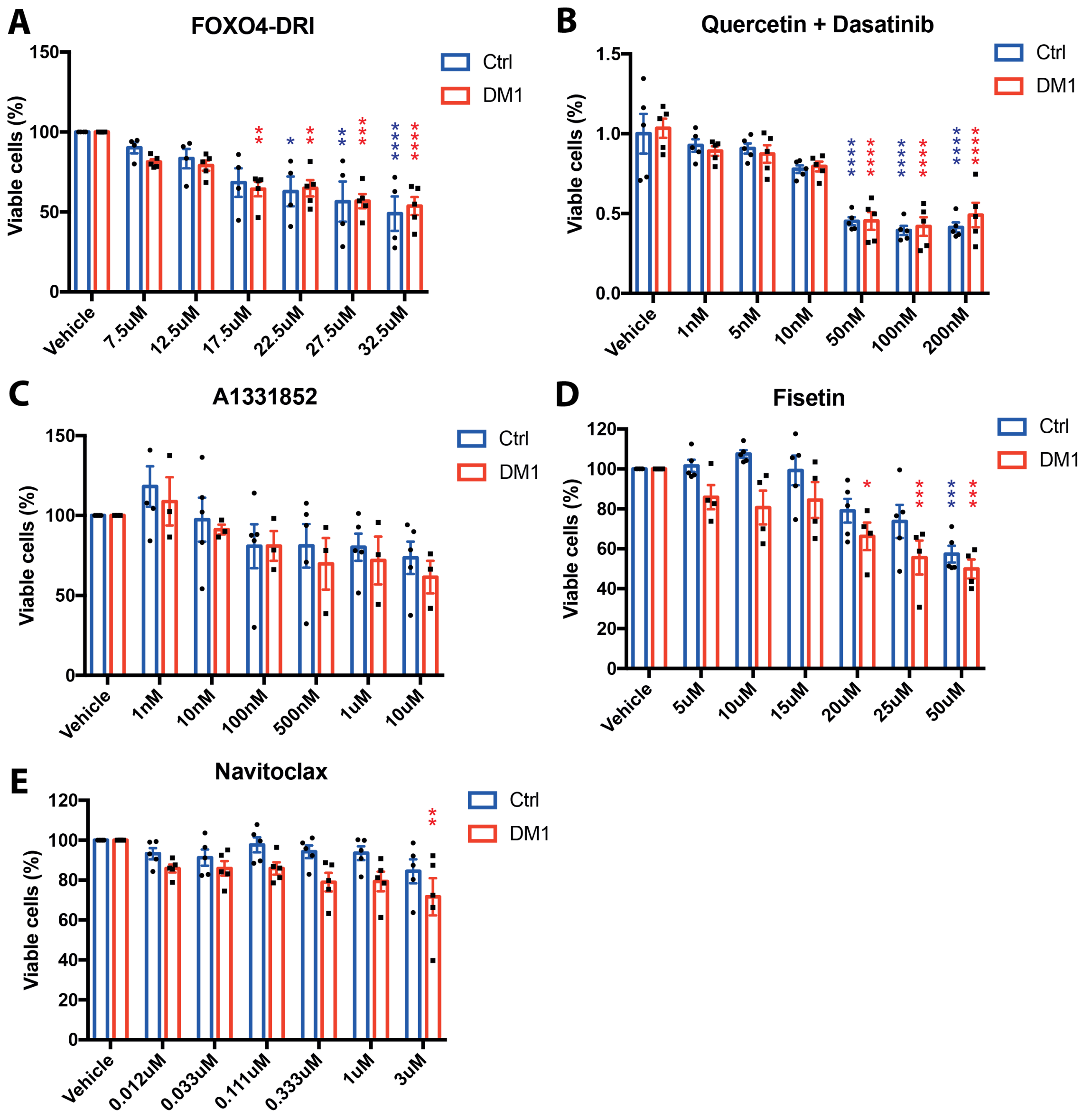

Supplemental Figure 8. **Senolytic drug screen**. Cell viability assay of control and DM1 myoblasts treated with different concentrations of: **A**) FOXO4-DRI (n=4 Ctrl and 5 DM1 cell lines), 17.5 µM: *p= 0.0056* (DM1); 22.5 µM: *p=0.0068* (DM1) and *p=0.0147* (Ctrl); 27.5 µM: *p=0.0002* (DM1) and *p=0.0015* (Ctrl), 32.5 µM: *p<0.0001* (DM1 and Ctrl), **B**) dasatinib + quercetin (n=5 Ctrl and DM1 independent cell lines), p<0.0001 (50 nM, 100 nM, and 200nM Ctrl and DM1), **C**) A1331852 (n=5 Ctrl and 3 DM1 independent cell lines), **D**) fisetin (n=5 Ctrl and 4 DM1 cell lines), 20 µM: *p= 0.025* (DM1); 25 µM: *p=0.0007* (DM1), 50 µM: *p<0.0001* (Ctrl and DM1); and **E**) Navitoclax (ABT-263, n=5 Ctrl and DM1 cell lines), 3 µM: *p=0.0013* (DM1). Data are expressed as means +/- SEM. 2-way ANOVA with Sidak's multiple comparisons test. *p<0.05, **p<0.01, ***p<0.001, ****p<0.0001. Red asterisks indicate significant difference compared to DM1 vehicle; blue asterisks indicate significant difference compared to Ctrl vehicle.

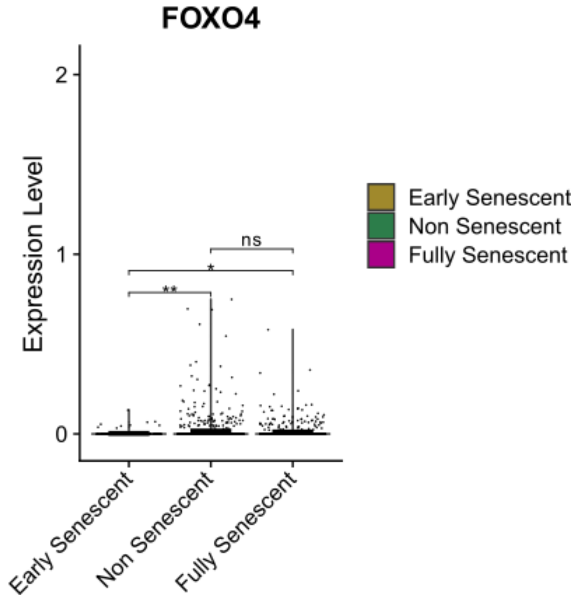

Supplemental figure 9. scRNAseq analysis of *FOXO4* expression. Violin plot showing the expression of *FOXO4* in fully senescent, early-senescent, and non-senescent subpopulations of DM1 myoblasts. NS= non-significant (p=0.48), *p=0.014, **p=0.0026 (One-sided Wilcoxon-Mann-Whitney unpaired U test).

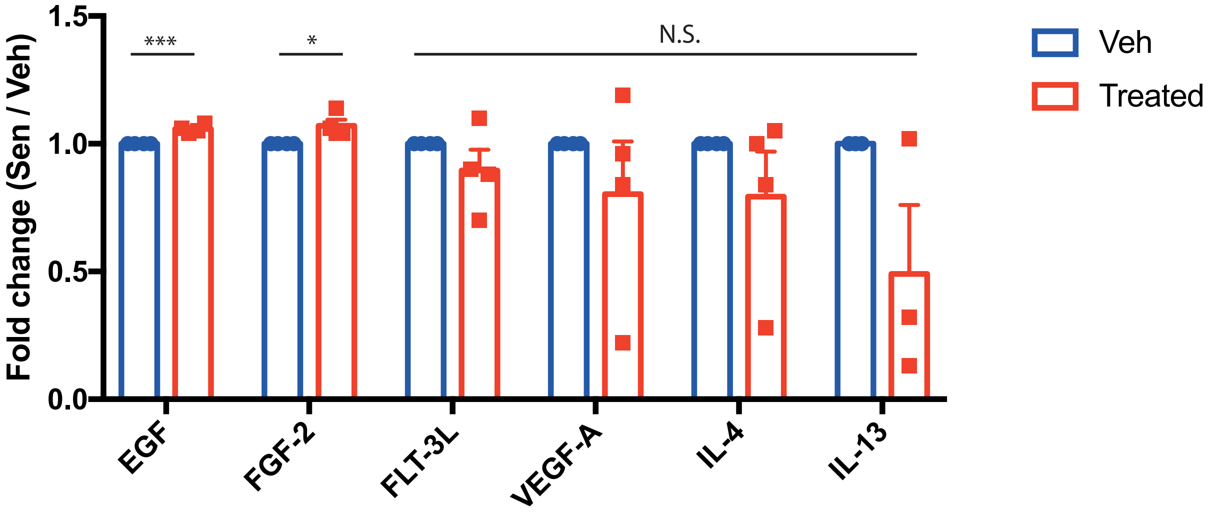

Supplemental Figure 10. Effect of senolytics on anti-inflammatory cytokines and growth factors. Multiplex Luminex assay of anti-inflammatory cytokines and growth factors markers found in the supernatant media of DM1 myoblasts treated or not with A1155463. Data are expressed as means +/- SEM. N=4 (except IL-13 treated group: n=3). p=0.0005 (EGF), p=0.046 (FGF-2), p=0.238 (FLT-3L), p=0.363 (VEGF-A), p=0.177 (IL-4), p=0.123 (IL-13). Multiple unpaired T-tests. N.S.: non-significant, *p<0.05, **p<0.01.

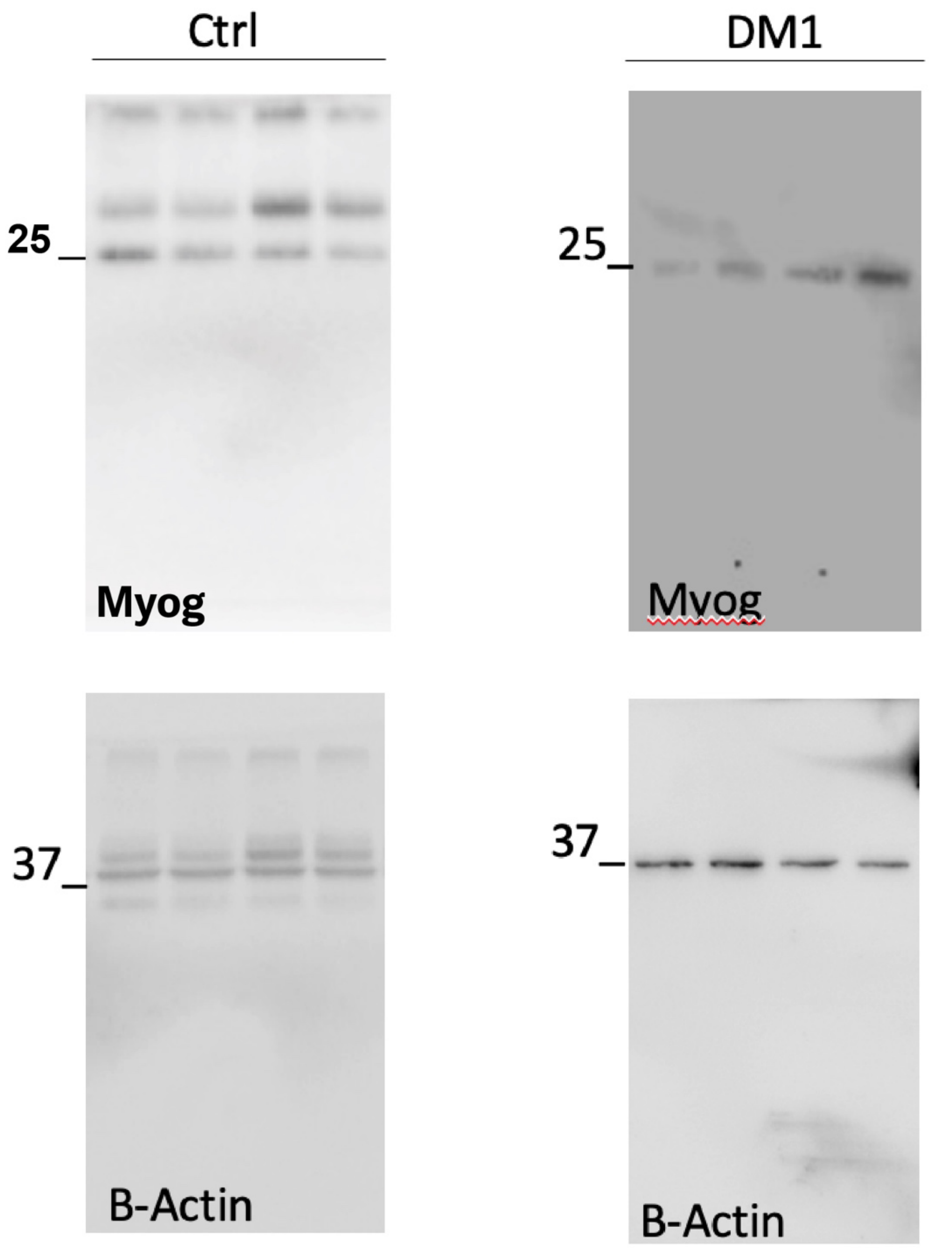

Supplemental Figure 11. Uncropped gels of Western blots. Full blots showing MYOG and β-actin staining (from Fig 6F).

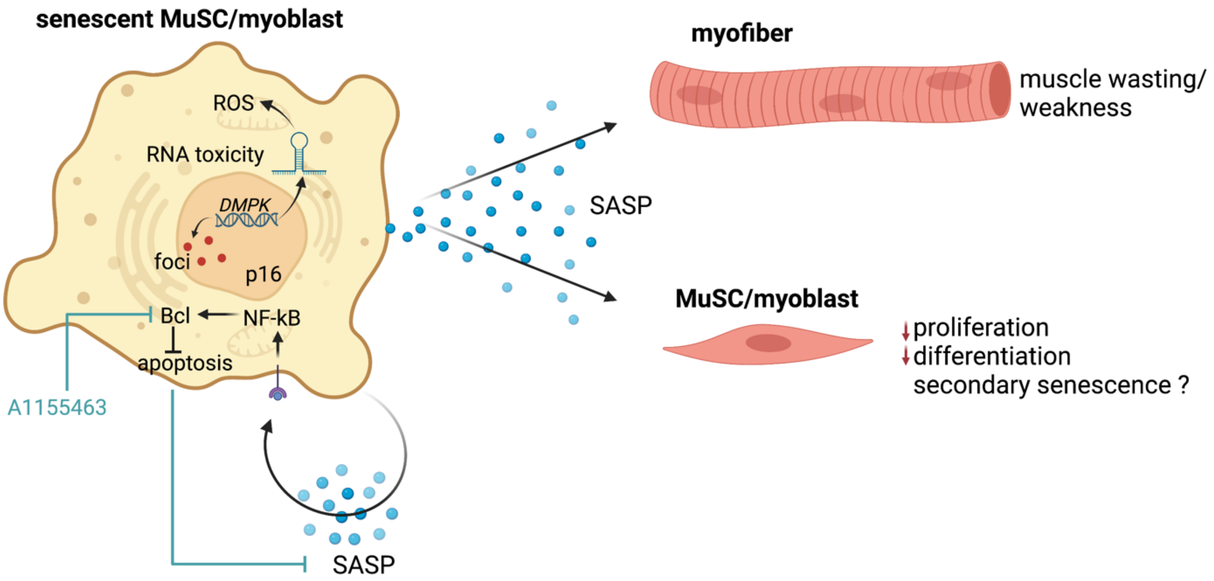

Supplemental figure 12. Schematic of the proposed mechanism. Expression of the mutated *DMPK* gene is associated with the accumulation of nuclear foci, RNA toxicity, ROS production and SASP. SASP acts as paracrine factors that have harmful effects on surrounding cells such as myofibers and muscle stem cells (MuSCs)/myoblasts. SASP activates NF-kB signalling, which in turn stimulates the activity of the anti-apoptotic Bcl family members. The senolytic drug A1155463 inhibits Bcl activity, thereby triggering the apoptosis of the senescent cell and reducing its expression of detrimental SASP. Created with BioRender.com

Participant number	CTG (rep)	Sex	Age (y)	scRNAseq
DM1-452	748	Woman	58	No
DM1-743	1097	Woman	51	No
DM1-1242	1200	Man	55	Yes
DM1-1594	673	Man	58	No
DM1-1621	675	Woman	36	No
DM1-1673	554	Woman	36	Yes
DM1-1692	954	Man	35	Yes
DM1-1751	1000	Woman	30	No
DM1-1770	800	Woman	32	No
DM1-1948	601	Woman	30	No
**Mean (SD)**	**830 (220)**		**42 (12)**	
CTR-4	N/A	Man	33	Yes
CTR-5	N/A	Woman	31	Yes
CTR-7	N/A	Woman	44	No
CTR-9	N/A	Woman	34	No
CTR-10	N/A	Man	45	No
CTR-11	N/A	Man	32	No
CTR-193	N/A	Man	50	Yes
**Mean (SD)**	**N/A**		**38.5 (7.5)**	

Supplemental table 1. Clinical characteristics of control and DM1 individuals that provided muscle biopsies. Abbreviations: CTG: cytosine-thymine-guanine; CTR: control subjects; DM1: myotonic dystrophy type 1 participants; rep: repetitions; y: years old; scRNAseq: single-cell RNA sequencing; SD: standard deviation.

**Characteristics**	**Total (n=103)**	**Low IL-6 level (n=80)**	**High IL-6 level (n=23)**
**Age, y**			
Mean (SD)	43.3 (10.1)	43.0 (10.7)	43.2 (7.8)
[min-max]	[20-77]	[20-77]	[30-69]
**Sex, n (%)**			
Men	38 (36.9)	29 (36.3)	9 (39.1)
Women	65 (63.1)	51 (63.7)	14 (60.9)
**Body mass index**			
Mean (SD)	25.4 (5.5)	24.1 (4.6)	28.7 (6.7)
[min-max]	[14.5-43.0]	[14.5-41.4]	[16.4-43.0]
**Phenotype, n (%)**			
Late-onset	20 (19.4)	17 (21.3)	3 (13.0)
Adult	83 (80.6)	63 (78.8)	20 (87.0)
**CTG repeat length**			
Mean (SD)	576.1 (378.1)	575.3 (399.5)	578.8 (299.8)
[min-max]	[59-2000]	[59-2000]	[63-1089]
**Disease duration (n = 74), y**			
Mean (SD)	19.7 (7.9)	19.5 (8.0)	20.3 (7.96)
[min-max]	[3-35]	[3-35]	[6-34]
**TNF-α level**			
Mean (SD)	1.67 (1.61)	1.68 (1.81)	1.63 (0.43)
[min-max]	[0.47-16.06]	[0.47-16.06]	[1.00-2.42]
**IL-6 level**			
Mean (SD)	3.28 (3.25)	2.04 (0.84)	7.61 (4.61)
[min-max]	[0.72-23.49]	[0.72-4.11]	[4.48-23.49]

Supplemental table 2. Clinical characteristics of DM1 patients that were subjected to functional capacity tests and assessment of serum IL-6 levels. y: years old, SD: standard deviation.

Test	R2, F value (df; Residual), p-value	Variables (Standardized B, p-value)
Timed-Up and Go	0.170, F (3, 99) = 6.77, p <.001	Phenotype (0.39, p<.001)<br>Age (0.35, p=.002)<br>**IL-6 (0.18, p=.048)**
10mWT	0.299, F (4, 98) = 10.46, p<.001	Phenotype (-0.48, p<.001)<br>Sex (-0.27, p=.002)<br>**IL-6 (-0.2, p=.003)**<br>Age (-0.23, p=.026)
Grip test	0.534, F (3, 96) = 36.68, p<.001)	Phenotype (-0.76, p<.001)<br>Sex (-0.30, p<.001)<br>Age (-0.19, p=.025)
Pinch test	0.492, F (2, 100) = 48.51, p<.001)	Phenotype (-0.64, p<.001)<br>Sex (-0.29, p<.001)
Ankle dorsiflexors	0.349, F (3, 98) = 17.49, p<.001)	Phenotype (-0.42, p<.001)<br>Sex (0.31, p<.001)<br>**IL-6 (-0.24, p=.005)**
Hip flexors	0.305, F (3, 98) = 14.34, p<.001)	Phenotype (-0.55, p<.001)<br>**IL-6 (-0.27, p=.002)**<br>Age (-0.32, p=.002)
Knee extensors	0.138, F (2, 99) = 7.95, p<.001)	Phenotype (-0.29, p=.003)<br>**IL-6 (-0.20, p=.034)**
Knee flexors	0.197, F (2, 100) = 12.23, p<.001)	Phenotype (-0.38, p<.001)<br>**IL-6 (-0.19, p=.034)**
Shoulder flexors	0.244, F (2, 97) = 15.65, p<.001)	Phenotype (-0.39, p<.001)<br>**IL-6 (-0.28, p=.002)**
Shoulder abductors	0.327, F (2, 98) = 23.76, p<.001)	Phenotype (-0.51, p<.001)<br>**IL-6 (-0.22, p=.009)**
Elbow flexors	0.347, F (2, 99) = 26.29, p<.001)	Phenotype (-0.55, p<.001)<br>**IL-6 (-0.18, p=.034)**
Elbow extensors	0.441, F (3, 98) = 19.09, p<.001)	Phenotype (-0.56, p<.001)<br>Sex (0.31, p<.001)<br>Age (-0.32, p<.001)<br>**IL-6 (-0.206, p<.001)**

Supplemental table 3. Step wise regression model of factors predicting muscle strength and functional outcomes in DM1 patients. 10mWT: 10-meter Walk test

Gene	Forward sequence	Reverse sequence
P16	TGTTCGCATTGCCAAGGTTC	CGTTTCCGTGAATGTTGTCCC
P21	CTGGGGATGTCCGTCAGAAC	GTGACAGGTCCACATGGTCT
RPLP0	GGCAGCATCTACAACCCTGA	CAGGACTCGTTTGTACCCGT

Supplemental table 4. List of primers used for qPCR experiments
