# Supplementary material for: Clearance of defective muscle stem cells by senolytics restores myogenesis in myotonic dystrophy type 1
Source: Nat Commun. 2023 Jul 19;14:4033. doi: 10.1038/s41467-023-39663-3 (PMC10356779; doi:10.1038/s41467-023-39663-3)
Supplement: Supplementary file 2 — Description of Additional Supplementary Files [file 41467_2023_39663_MOESM2_ESM.pdf]

Description of Additional Supplementary Files

Supplementary Data 1: List of differentially expressed genes (DEG) between control and DM1 patients myoblasts showing the p-value (p_val), the average log2 fold-change (avg_log2FC), the proportion of cells expressing each gene in control and patients, and the adjusted p-value (adj_p_val). Wilcoxon rank-sum test with the Benjamini-Hochberg procedure for False-discovery rate (FDR) control.
